# Factors associated with substance use disorder treatment completion: a cross-sectional analysis of justice-involved adolescents

**DOI:** 10.1186/s13011-020-00332-z

**Published:** 2020-12-07

**Authors:** Micah E. Johnson, Dieu X. Tran

**Affiliations:** 1 Department of Mental Health Law and Policy, College of Behavioral and Community Sciences, University of South Florida, 13301 Bruce B. Downs Blvd, Tampa, FL 33612 USA; 2 Department of Epidemiology and Biostatistics, College of Public Health, University of South Florida, 13201 Bruce B. Downs Blvd, MDC 56, Tampa, FL 33612 USA

**Keywords:** Justice-involved adolescents, Substance use disorder, Treatment adherence, Substance misuse

## Abstract

**Background:**

Substance use disorders (SUD) are prevalent among those in the juvenile justice system. SUD treatment programs implemented in correctional settings can prevent overdose and other health-related problems among an underserved health disparity population. However, only a fraction of justice-involved adolescents with SUDs complete a treatment program and the factors associated with treatment completion among adolescents in the criminal justice system have not been thoroughly investigated.

**Methods:**

Using cross-sectional data on 25,587 adolescents from the Florida Department of Juvenile Justice (FLDJJ) who met the criteria for SUD treatment, the study investigated the factors associated with the completion of SUD treatment. Sociodemographic, mental health, and other variables were examined.

**Results:**

Several factors were associated with an increased likelihood of SUD treatment completion: previous participation in treatment programs, prior drug and alcohol education class attendance, and involvement in court-directed programs. Additional factors included multiple incarcerations, and strong financial and support networks.

**Conclusions:**

The strongest factors associated with a higher likelihood of SUD treatment completion among adolescents in the justice system are ones that can be translated into programs and practices. Repeated referrals to treatment, court-directed programs, and strong support networks may yield higher rates of completion.

## Background

Substance use (SU) and substance use disorders (SUDs) that emerge during adolescence are associated with high morbidity and mortality, along with several adverse consequences such as risky behaviors, delinquency, and recidivism [1–4]. In the United States, rather than entering a behavioral healthcare system, many adolescents are inappropriately siphoned into the criminal justice system as it has become synonymous with substance misuse treatment systems. Previous studies have observed higher rates of SUDs among the juvenile justice system compared to the general population [1, 5]. The effects of untreated SUDs can be more deleterious for justice-involved adolescents (JIA) who are associated with diverse risk factors. The circumstances and collateral consequences of justice involvement can exacerbate the negative effects of untreated SUDs on their health, behavior, and community.

Many adolescents access behavioral healthcare for the first time through the juvenile justice system [2]. Some individuals are mandated by the court to attend a substance misuse education course or a SUD treatment program [1, 6]. For many adolescents, incarceration is a rare opportunity to receive accessible treatment in a relatively stable setting [1, 2, 6]. Completion of SUD treatment can result in worthwhile individual and community outcomes [1, 7]. Despite the damages associated with untreated SUDs, many JIA who are referred to treatment do not complete their assigned treatment programs [1]. Failure to adhere to an SUD treatment program can lead to relapse and re-arrest during the first 12 months of release [2, 8]. These alarming trends underscore the need to pinpoint the factors that increase the likelihood of treatment completion among JIA [2]. Although the needs of JIA extend beyond SUD treatment to include vocational, legal, family, educational, mental health, and healthcare components, treatment adherence is a critical step towards improving the health of these adolescents, their families, and their communities [1, 9].

### SUD treatment completion

Court-ordered interventions and sanctions have been associated with increased participation in SUD treatment programs among adolescents [1, 9]. Coerced or involuntary SUD treatment for JIA have shown to reduce nationwide juvenile substance misuse and related arrests, overdoses, and deaths [4, 10]. Low retention and high early dropout rates were exhibited despite efforts made to enroll JIA into SUD treatment programs [11]. Understanding the characteristics that contribute to SUD treatment completion among JIA can promote public safety and reduce relapse, overdose, and early death [1, 5].

Among adolescents, sociodemographic factors such as race and gender exhibited significant roles in SUD treatment completion [12, 13]. Previous studies reported poor treatment completion outcomes in the presence of gender inequalities and racial disparities [12–14]. Additionally, many investigations identified the relationship between mental health conditions, such as anxiety and depression, and low SUD treatment completion [5, 7, 15, 16]. Childhood trauma influences SUD treatment completion through psychological distress and trauma-related cognitions [17]. JIA exposed to higher levels of trauma were associated with increased risk for relapse and presented minimal treatment adherence [17].

Addressing the needs of psychiatric disorders by establishing strong support networks and fostering optimism has demonstrated higher rates of SUD treatment completion [1, 18]. Support networks (e.g. romantic partners, social peers, and family support) have been shown to produce greater levels of self-confidence, concentration, and motivation [19, 20]. Likewise, optimism stimulates several positive health and behavioral outcomes [21]. Despite these findings, other known factors associated with SUD treatment completion in juvenile justice systems have not been well studied.

### The current study

In this study, we aimed to identify the factors that were associated with SUD treatment completion among adolescents in the juvenile justice system. We explored sociodemographic elements such as financial distress and support networks. We hypothesized that higher levels of social support would be associated with higher odds of SUD completion compared to less social support. We also hypothesized that family history of SUD and low socioeconomic status may create barriers that lower the odds of treatment completion [22].

Additional factors that were examined include school enrollment, previous participation to SUD treatment programs, previous drug and alcohol class attendance, and history of criminal offenses. We predicted that lack of full-time school enrollment associated with low treatment adherence may subsequently lower the odds of SUD treatment completion [23]. Prior participation in treatment programs may have higher odds of treatment completion attributable to motivation, confidence, and other factors related to recovery capital [24, 25]. Individuals with a past criminal history may also have higher odds of treatment completion due to motivation to avoid harsher penalties and access to a structured treatment delivery environment [24, 26]. The hypotheses were tested using statewide data from Florida Department of Juvenile Justice (FLDJJ).

## Methods

### Data

The study uses data from FLDJJ, a state agency of Florida that manages juvenile justice and operates juvenile detention centers. Since 2004, FLDJJ has collected data on all individuals who are arrested using a comprehensive assessment and case management process. When a minor is arrested in Florida, an enrollment process is completed to enter the FLDJJ system. As a part of the enrollment process, all children are administered the Positive Achievement Change Tool (PACT) assessment. This risk assessment tool was developed and administered by federal agencies to predict an adolescent’s likelihood to re-offend based off of known risk factors with 95% confidence intervals [27]. The variables measured in the PACT resemble the variables investigated in this study. The PACT instrument has been validated across multiple samples of FLDJJ data published in several peer-reviewed journals (see 27). Trained personnel conduct semi-structured interviews using the PACT software. The interface guides all aspects of data collection; it includes open-ended questions, an interview guide, the PACT manual and coding techniques. Each adolescent in the sample has a unique identifier that helps prevent double registration. This report includes 80,441 JIA in the FLDJJ PACT dataset; < 1% (*n* = 481) of the total cases were omitted due to missing data on SU, resulting in a final dataset of 79,960 JIA.

The sample of 79,960 represents all children who entered the FLDJJ from 2004 to 2015, completed the Full PACT assessment, and reached the age of 18 by year 2015. Out of this sample, 25,587 incarcerated children met the criteria for a SUD diagnosis. Nearly 41.1% were non-Latinx White (*n* = 10,512), 42.9% of subjects were non-Latinx Black or African American (n = 10,981), 15.5% were Latinx (*n* = 3975), and 0.5% were another race (*n* = 119). Roughly 17.5% of the sample were female (*n* = 4478) and the mean age at the initial assessment was 14.

### Measures

#### SUD treatment completion

The construct lifetime treatment completion refers to the completion of the prescribed SUD treatment program. The dichotomous measure coded JIA as 0, “never completed SUD treatment program” and 1 “met FLDJJ evidence criteria for completion of a SUD treatment program.” FLDJJ evidence criteria for completion include official documentation from a credentialed or licensed treatment provider, such as a licensed physician, counselor, or other behavioral healthcare professional, that the individual successfully completed the treatment program.

#### Criminal history

Criminal history refers to the existence of a criminal record stored in a repository of criminal history records. Detention placement history was measured via four-item ordinal variable reporting the number of previous holdings in a juvenile justice facility. Response items were (0) none, (1) one, (2) two, (3) three or more. Commitment placement history was measured via three-item ordinal variable reporting the number of previous incarcerations. Response items were (0) none, (1) one, or (2) two or more. Previous participation in treatment programs was measured through a three-item ordinal variable. Response items were (0) none, (1) once participated in a program, (2) participated multiple times in programs. Attendance in previous drug and alcohol courses was measured through four-item nominal variable: (0) never participated in program, (1) voluntary attendance, (2) attendance by request, (3) assigned by court-direction. Drug use was recorded through a four-item nominal measure: (0) Alcohol only, (1) marijuana only, (2) alcohol and marijuana only, (4) hard drugs. Data were collected through PACT interviews and coded by trained FLDJJ staff.

#### Trauma

Adverse childhood experiences (ACEs) are potentially traumatic events that occur in childhood such as experiencing or witnessing violence, neglect, abuse, or death. There were 11 categories of childhood trauma that were evaluated through a dichotomous measure *(0 = no this did not occur, 1 = yes this did occur).* The 11 dichotomous measures of childhood trauma were summed to create an additive cumulative trauma index ranging from 0 to 11 types of trauma; each type of trauma counted as one. This study examined the effects of one type of trauma: witnessing violence. There are many types of trauma, however violence was most commonly reported in the ACE instrument by FLDJJ. Runaway or kicked out of home status was measured through a three-item nominal variable. Response items were (0) none, (1) has ran away or been kicked out of home, and (2) is currently a runaway or kicked out of home. Data were collected through PACT interviews and coded by trained FLDJJ staff.

#### School enrollment

Current enrollment status referred to the adolescent’s middle or high school enrollment status. It was operationalized through a six-item categorical variable reporting enrollment at intake. Response items were (0) graduated or GED, (1) enrolled full-time, (2) enrolled part-time, (3) suspended, (4) dropped out, or (5) expelled. The likelihood of staying in school to obtain graduate or GED status was assessed through a three-item ordinal measure: (0) not likely, (1) uncertain, (2) very likely. Data were collected through PACT interviews and coded by trained FLDJJ staff.

#### Support network

The construct of a support network refers to a group of people who provide emotional, financial, and practical aid to someone who presents situational difficulties. In this investigation, the group of people selected were immediate family members to the participant. Level of support was assessed via three-item ordinal measure. Response items were (0) none, (1) some support network, (2) strong support network. Admiration of anti-social peers refers to the respect or approval towards peers who exhibit anti-social behavior. Anti-social behavior includes hostility or disruption to legal social order. The variable was operationalized with a three-item ordinal measure. Response items were (0) does not admire anti-social peers, (1) somewhat admires anti-social peers, and (2) admires anti-social peers. Family income was measured via a four-item ordinal variable reporting the combined annual income of the adolescent’s family members. Response options were (0) under $15,000, (1) from $15,000 to $34,999, (2) from $35,000 to $49,999, and (3) $50,000 and above. Data were collected through PACT interviews and coded by trained FLDJJ staff.

#### Optimism

Current optimism was measured by an ordinal variable reporting the adolescent’s level of optimism. The measure was reverse coded such that higher values represented lower levels of optimism. Response items were (0) high optimism, (1) normal optimism, (2) low optimism, and (3) very low optimism. High optimism indicated high aspirations, sense of purpose and committed to better life. Normal optimism indicated normal aspirations and sense of purpose. Low optimism indicated low aspirations and little sense of purpose or plans for better. Very low optimism indicated that the individual believed nothing matters and expected to be dead soon. Data were collected through PACT interviews and coded by trained FLDJJ staff.

#### Control variables

The study adjusts for known factors of current (past six month) SUD (gender, race, age, and history of mental diagnosis). Gender is a social construct that includes the sex categorizations male and female. Gender is widely acknowledged by sexual minorities as being less offensive and more inclusive and thus is used in place of sex to refer to males and females. Female gender was operationalized by a self-reported dichotomous measure (0 = *male gender*, 1 = *female gender*). Race is a social construct that often includes or is used synonymously with ethnicity and national origin. Therefore, race was used to refer to race and ethnicity. Race was operationalized via a four-item nominal measure (0 = *White*, 1 = *Black*, 2 = *Latinx*, 3 = *other*). Age was operationalized via a continuous variable ranging from 18 to 26 in 2015.

History of mental health diagnosis was measured via a dichotomous variable reporting the history of a mental health diagnosis at intake. Response items were (0) no history of mental health diagnosis or (1) diagnosed with mental health problems. Furthermore, the number of mental health diagnoses was recorded via a three-item ordinal measure *(0 = none, 1 = one, 2 = two or more)*. In the data, FLDJJ researchers combined depression and anxiety [28]. History of depression/anxiety was measured via an ordinal variable reporting the history of depression and/or anxiety at intake. Response items were (0) no history of depression/anxiety, (1) occasional depression, (2) consistent depression/anxiety but no impairment, (3) impairment from consistent depression.

### Analytical procedures

Data analysis was conducted using STATA, version 15 SE [29]. A complete case analysis was appropriate and there was minimal missing data (< 1%) that was assumed Missing Completely at Random (MCAR). The sample size was 25,587. Demographic data were summarized using descriptive statistics. A chi-square test of independence was performed to compare whether there was a significant association between categorical variables and the likelihood of SUD treatment completion. An independent t-test was conducted to compare the means of the interval variable (age) between non-SU versus SU. Multivariate logistic regression was used to calculate adjusted odds ratios (aORs) and 95% confidence intervals for SUD treatment completion. The covariates of gender, race, age, annual household income, ACE measure score, drug use, mental health diagnoses, history of treatment program participation, history of drug and alcohol classes, history of witnessing violence, runaway or kicked out status, detention placement history, commitment placement history, school enrollment, likelihood of school completion, admiration of anti-social peers, support network, and optimism were controlled in the multivariate model. The probability and the marginal log odds were estimated (using the STATA margins procedures) and graphically display the data. The Hosmer-Lemeshow test was used to confirm adequate model fit.

## Results

### Univariate

Within the total sample of 79,960 JIA, the univariate analysis described the sample of 25,587 JIA, which met the criteria for SUD. Among the group, an average age of 14 years was observed at initial intake. Over 58% of the sample were individuals of color and around 83% of the sample was composed of males. Nearly 70% of participants reported an annual household income of $34,999 or lower. About 59% of JIA were not diagnosed with a mental health condition and almost 79% of JIA have a history of witnessing violence. Approximately 49.8% of the sample reported normal levels of optimism. More than 54.2% have never participated in a drug and alcohol treatment program and 49.8% of JIA have never attended a drug and alcohol education class. Additionally, 45.2% of JIA were uncertain of continuing high school education and 42.7% of JIA withdrew from high school.

### Bivariate

In the sample of 25,587 JIA, approximately 88.5% JIA did not complete SUD treatment. About 65.7% of JIA who have completed SUD treatment had a reported annual household income less than $34,999. Roughly 20.3% of JIA who did not complete treatment earned an annual household income under $15,000. Nearly 47.5% of JIA who completed SUD treatment were white compared to the 34.9% of JIA who were Black. 87.9% of individuals who completed SUD treatment had at least one mental health diagnosis. Those who completed SUD treatment were associated with taking drug and alcohol classes at court direction (54.3%) while individuals who did not complete SUD treatment were associated with no history of drug and alcohol classes (49.8%). Only 8.2% of JIA who completed SUD treatment were associated with high levels of optimism compared to those who did not (5.9%). JIA who did not complete treatment showed a higher percentage of low optimism (1.8%) compared to the completed group (1.6%). Table 1 and Table 2 display descriptive statistics and bivariate analyses. Results from the Spearman’s Correlation Matrix show several negligible and weak relationships between the variables and treatment completion. Multiple mental health diagnoses, previous multiple participations in treatment programs, and previous drug and alcohol class attendance had weak positive associations with treatment completion. See Table 3.

**Table 1:** Characteristics of JIA in Current SUD Treatment Programs

	Overall (*n* = 25,587)	%	Not Completed (*n* = 22,634)	%	Completed (*n* = 2953)	%
	n		n		n	
Race						
White	10,512	41.1	9109	40.2	1403	47.5
Black	10,981	42.9	9.951	44	1030	34.9
Latinx	3975	15.5	3466	15.3	509	17.2
Other	119	0.5	108	0.5	11	0.4
Gender						
Male	21,109	82.5	18,603	82.2	2506	84.9
Female	4478	17.5	4031	17.8	447	15.1
Household Income						
Under $15 k	4991	19.5	4587	20.3	404	13.7
From $15 k to $34,999	13,817	54	12,282	54.3	1535	52
From $35 k to $49,999	4519	17.7	3889	17.2	630	21.3
$50 k & Over	2198	8.6	1819	8	379	12.8
Age^+^mean (SD)	14.07 (3.75)					
Drug Use						
Alcohol Only	2702	10.6	2139	9.5	563	19.1
Marijuana Only	7486	29.3	6820	30.1	666	22.6
Alcohol and Marijuana Only	9243	36.1	8300	26.7	943	31.9
Hard Drugs	6156	24.1	5375	23.7	781	26.4
Mental Health Diagnoses						
None	15,311	59.8	14,278	63.1	1033	35
One	8.542	33.4	6981	30.8	1561	52.9
Two	1734	6.8	1375	6.13	359	12.2
History of Participation in a Treatment Program						
Never	13,879	54.2	13,352	59	527	17.8
Once	8887	34.7	7188	31.8	1699	57.5
Multiple Times	2821	11	2094	9.3	727	24.6
History of Attending Drug and Alcohol Classes						
Never	12,753	49.8	12,253	54.1	502	17
Voluntarily Attended	1368	5.3	1162	5.1	206	7
Attended by Request	3770	14.7	3128	13.8	642	21.7
Attended at Court Direction	7696	30.1	6093	26.9	1603	54.3
ACE Measure Summary Score^+^mean (SD)	4.14 (2.04)					

Chi square statistics: Race (Χ^2^ = 90.65, p = 0.000); gender (Χ^2^ = 12.92, p = 0.000); household income (Χ^2^ = 156.82, *p* = 0.000); age (Χ^2^ = 77.39, *p* = 0.000); drug use (Χ^2^ = 304.10, *p* = 0.000); mental health diagnoses (Χ^2^ = 866.66, *p* = 0.000); history of participation (Χ^2^ = 1874.52, *p* = 0.000); history of attending classes (Χ^2^ = 1500.54, p = 0.000); ACE measure (Χ^2^ = 57.32, p = 0.000); history of witnessing violence (Χ^2^ = 2.01, *p* = 0.157); support network (Χ^2^ = 259.60, p = 0.000); runaway (Χ^2^ = 6.81, *p* = 0.033); detention placement (Χ^2^ = 24.08, p = 0.000); commitment placement (Χ^2^ = 226.92, p = 0.000); admiration of social peers (Χ^2^ = 9.21, *p* = 0.010); school enrollment (Χ^2^ = 54.17, p = 0.000); likelihood of staying in school (Χ^2^ = 224.75, p = 0.000); optimism (Χ^2^ = 41.17, 0.000)

**Table 2:** Characteristics of JIA in Current SUD Treatment Programs Continued

	Overall (*n* = 25,587)		Not Completed (*n* = 22,634)		Completed (*n* = 2953)	
	n	%	n	%	n	%
History of Witnessing Violence						
No	5351	20.9	4704	20.8	647	21.9
Yes	20,236	79.1	17,930	79.2	2306	78.1
Family Support Network						
None	1791	7	1688	7.5	103	3.5
Some	13,459	52.6	12,198	53.9	1261	42.7
Strong	10,337	40.4	8748	38.6	1589	53.8
Runaway/Kicked Out History						
Never	13,988	54.7	12,324	54.4	1664	56.3
Has Ran Away/Been Kicked Out	10,516	41.1	9330	41.2	1186	40.2
Current Runaway or Kicked Out	1083	4.2	980	4.3	103	3.5
Secure Detention Placement History						
None	6057	23.7	5358	23.7	699	23.7
One	5645	22.1	5073	22.4	572	19.4
Two	4362	17	3885	17.2	477	16.2
Three or More	9523	37.2	8318	36.8	1205	40.8
Commitment Placement History						
None	14,419	56.4	13,110	57.9	1309	44.3
One	8503	33.2	7331	32.4	1172	39.7
Two or More	2665	10.4	2193	9.7	472	16
Admiration of Anti-Social Peers						
Does Not Admire	5161	20.2	4518	20	643	21.8
Somewhat Admires	13,892	54.3	12,279	54.3	1613	54.6
Admires	6534	25.5	5837	25.8	587	23.6
Enrollment Status						
Graduated or GED	274	1.1	226	1	48	1.6
Enrolled Full-Time	8307	32.5	7246	32	1061	35.9
Enrolled Part-Time	1012	4	868	3.8	144	4.9
Suspended	1496	5.8	1301	5.7	195	6.6
Dropped Out	10,927	42.7	9818	43.4	1109	37.6
Expelled	3512	13.7	13.7	13.8	388	13.1
Likelihood of Staying in School						
Not Likely	4388	17.1	3703	16.4	685	23.2
Uncertain	11,554	45.2	10,169	44.9	1385	46.9
Very Likely	5898	23.1	5207	23	691	23.4
Optimism Level						
High aspirations	1572	6.1	1329	5.9	243	8.2
Normal aspirations	12,737	49.8	11,201	49.5	1536	52
Low aspirations	10,822	42.3	9694	42.8	1128	38.2
Believes nothing matters	456	1.8	410	1.8	46	1.6

Data displayed as column percent. Symbol “+” signifies an interval variable and the mean is reported with the standard deviations (SD) in parentheses. *N* = 25,587

**Table 3:** Spearman’s Correlation Matrix for All Variables Continued

	11	12	13	14	15	16	17	18	19
11. History of Witnessing Violence	–								
12. Family Support Network	0.03^***^	–							
13. Runway/Kicked Out History	0.09^***^	−0.02^**^	–						
14. Secure Detention Placement History	0.23^***^	0.05^***^	0.26^***^	–					
15. Commitment Placement History	0.16^***^	0.17^***^	0.20^***^	0.45^***^	–				
16. Admiration of Anti-Social Peers	0.14^***^	−0.02^***^	0.21^***^	0.25^***^	0.23^***^	–			
17. School Enrollment	−0.04^***^	−0.02^***^	− 0.01	−0.03^***^	− 0.01^*^	−0.03^***^	–		
18. Likelihood of staying in school	−0.05^***^	−0.18^***^	0.00	−0.08^***^	− 0.16^***^	−0.06^***^	0.12^***^	–	
19. Optimism Level	0.12^***^	−0.11^***^	0.20^***^	0.23^***^	0.22^***^	0.37^***^	−0.00	−0.00	–

^*^ *p* < 0.05, ^**^ *p* < 0.01, ^***^ *p* < 0.001

### Multivariate

The multivariate logistic regression model in Table 4 displays variables related to the likelihood of SUD treatment completion. The model included: gender, race, age, annual household income, ACE measure score, drug use, mental health diagnoses, history of treatment program participation, history of drug and alcohol classes, history of witnessing violence, runaway or kicked out status, detention placement history, commitment placement history, school enrollment, likelihood of school completion, admiration of anti-social peers, support network, and optimism. The variables in the multivariate model that were significantly associated with high increased odds for SUD treatment completion were previous participation in a treatment program and previous drug and alcohol class attendance, specifically at court direction or at the request of friends, family, or an agency.

**Table 4:** Logistic Regression Estimating Substance Use Treatment Completion

	AOR	CI
Female (Ref = Male)	0.94	[0.83,1.06]
Black (Ref = White)	0.85^**^	[0.76,0.94]
Latina/o	1.02	[0.91,1.15]
Other	0.82	[0.42,1.58]
From $15,000 to $34,999 (Ref = Under $15000)	1.08	[0.95,1.22]
From $35,000 to $49,999	1.13	[0.97,1.31]
$50,000 & over	1.31^**^	[1.10,1.56]
Age	0.91^***^	[0.90,0.93]
MJ Only (Ref = AL Only)	0.36^***^	[0.32,0.42]
AL & MJ Only	0.39^***^	[0.34,0.44]
Hard Drugs	0.41^***^	[0.36,0.48]
Mental Health Diagnosis (Ref = None)	1.24^***^	[1.12,1.37]
Two Or More	1.35^***^	[1.16,1.58]
Once participated in a treatment program (Ref = None)	3.37^***^	[2.89,3.92]
Participated multiple times in treatment programs	4.72^***^	[3.96,5.63]
Voluntarily attended drug or alcohol education classes (Ref = None)	1.54^***^	[1.25,1.89]
Attended class at request	1.86^***^	[1.57,2.21]
Attended classes at court direction	2.17^***^	[1.84,2.55]
ACE Measure Summary Score	0.95^***^	[0.93,0.98]
History of witnessing violence (Ref = None)	0.89^*^	[0.80,1.00]
Some Support Network (Ref = None)	1.16	[0.93,1.44]
Strong Support Network	1.36^**^	[1.09,1.70]
Has run away or been kicked out of home (Ref = None)	0.85^***^	[0.77,0.93]
Is currently a runaway or kicked out of home	0.75^*^	[0.60,0.95]
Detention Placement History (Ref = None)	0.83^**^	[0.73,0.95]
Two	0.84^*^	[0.73,0.97]
Three or more	0.85^*^	[0.75,0.97]
Commitment placement history (Ref = None)	1.06	[0.96,1.18]
Two or more	1.26^**^	[1.09,1.46]
Somewhat admires or emulates anti-social peers (Ref = Does Not)	0.96	[0.86,1.08]
Admires or emulates anti-social peers	0.83^*^	[0.72,0.96]
Enrolled Full-Time (Ref = Graduated or GED)	0.55^**^	[0.38,0.79]
Enrolled Part-Time	0.57^**^	[0.38,0.85]
Suspended	0.58^**^	[0.39,0.86]
Dropped Out	0.49^***^	[0.34,0.70]
Expelled	0.50^***^	[0.35,0.74]
Likelihood of staying in school (Ref = Very Likely)	1.06	[0.43,2.57]
Uncertain to stay in school & graduate	0.81^***^	[0.72,0.91]
Not very likely to stay in school & graduate	0.84^*^	[0.73,0.97]
Optimism (Ref = High)	0.42^***^	[0.34,0.50]
Normal aspirations	0.83^*^	[0.70,0.98]
Low aspirations	0.72^***^	[0.60,0.87]
Believes nothing matters	0.68^*^	[0.47,0.98]
Constant	1.01	[0.57,1.79]
Observations	25,587	

95% confidence intervals in brackets ^*^ *p* < 0.05, ^**^ *p* < 0.01, ^***^ *p* < 0.001

JIA who have participated one drug and alcohol treatment program were 3.37 times higher odds of completing SUD treatment as JIA who have not participated in a treatment program (aOR: 3.37; 95% CI 2.89, 3.92). Those who have engaged in multiple treatment programs previously were 4.72 higher odds of completing SUD treatment (aOR: 4.72; 95% CI 3.96, 5.63) Correspondingly, voluntary participation in drug and alcohol classes by the individual showed 54% increased odds (aOR: 1.54; 95% CI 1.25, 1.89). Meanwhile, those who attended classes at the request of friends, family, or an agency demonstrated 86% increased odds (aOR: 1.86; 95% CI 1.57, 2.21). JIA attending classes at court direction had 2.17 times higher odds to complete treatment compared to individuals who did not (aOR: 2.17; 95% CI 1.84, 2.55). Having a history of two or more commitment placements showed 26% increased odds of SUD treatment completion (aOR: 1.26; CI 1.09,1.46).

JIA with strong support network levels had 36% higher odds to complete treatment compared to JIA with no support network (aOR: 1.36; 95% CI 1.09, 1.70). Those who admire or emulate their anti-social peers had lower odds of completing SUD treatment (aOR: 0.83; 95% CI 0.72, 0.96). Individuals who have reported normal levels of optimism presented lower odds compared to individuals with high levels of optimism (aOR: 0.83; 95% CI 0.70, 0.98). As optimism levels decrease, individuals with low aspirations and little sense of purpose had 28% lower odds (aOR: 0.72; 95% CI 0.60, 0.87). JIA who believe nothing matters and expect to be dead soon had 32% lower odds of completing SUD treatment (aOR: 0.68; 95% CI 0.47, 0.98).

JIA with a reported annual household income above $50,000 had 1.31 times completing SUD treatment as those with an income under $15,000 (aOR: 1.31 95% CI 1.10, 1.56). Compared to White JIA, Black JIA had lower odds of completing SUD treatment (aOR: 0.85; CI 0.76, 0.94). Individuals diagnosed with one mental health diagnosis had 24% increased odds of completing SUD treatment compared to no mental health diagnosis (aOR: 1.24; 95% CI 1.12, 1.37). Accompanied by an increase of mental health diagnoses, individuals with two or more mental health diagnoses presented a 35% increased odds of SUD treatment completion (aOR: 1.35; CI 1.16, 1.58).

## Discussion

### Synopsis

Using statewide data from FLDJJ, the associations between sociodemographic factors and SUD treatment completion were investigated with a sample of 25,587 JIA. Our findings indicate previous participation in treatment programs, previous drug and alcohol class attendance, history of multiple commitment placements, court-directed programs, strong financial and emotional support networks, and high levels of optimism had increased likelihood of SUD treatment completion.

### Implications

Though 32% of JIA met the criteria for SUD treatment, only 11.5% of the sample completed a SUD treatment program. The rate of completion is remarkably lower than the reported rate in the National Survey of Drug Use and Health (NSDUH). A low treatment completion rate may be associated with multiple factors such as health disparities, unmet SUD treatment completion needs, and continuity of SUD intervention programs.

Within the juvenile justice system, health disparities manifest from differential treatment among offenders of color [12, 13]. In this study, the distribution of JIA of color to white JIA were roughly equal yet JIA of color presented lower rates of completion. JIA of color also had slightly lower odds of completing SUD treatment. Through differential treatment, unmet SUD treatment completion needs may arise [12].

In this investigation, previous participation in a treatment program yielded an increased rate of SUD treatment completion when compared with no previous participation. With increased exposure to treatment programs, the likelihood of SUD treatment completion rises. This may suggest the mechanism of reinforcement, which contributes to higher levels of motivation [22, 25]. The results demonstrate higher rates of completion among those who attend at court-direction compared to voluntarily attended and among those with multiple incarcerations compared to none. JIA with a history of multiple incarcerations may view court-directed programs as a prospective substitute to extended imprisonment or severe penalties [24, 30]. Furthermore, in some cases, court-directed programs may be regarded as more accessible routes to SUD treatments [22].

Individuals who could not previously seek SUD treatment in the past due to financial distress may see court-directed programs as an opportunity to receive treatment [22, 31]. However, those who choose not to participate in treatment programs may believe it is not worth beginning if it may be difficult to obtain continuity of care after program release due to financial stress [1]. Moreover, the results display a higher likelihood of treatment completion among those with an annual household income above $50,000. Financial distress has the potential to reduce program enrollment and may inhibit an individual from completing treatment, fueling the continuity of addictive behavior and criminal activity [22, 32].

Alongside financial support, having a strong support network is a significant factor in SUD treatment completion [25, 32]. The relation between positive emotional support and treatment completion implies the potential role optimism, admiration of anti-social peers, and support networks may contribute [20]. Self-stigma and general public stigma regarding substance use may impact an individual’s behavior towards seeking help and achieving remission [20]. Stigmatizing attitudes are pervasive and subjects one to feelings of isolation and rejection [19]. In this investigation, individuals are more likely to complete SUD treatment programs when there were remarkably stronger support networks and less admiration towards anti-social peers. JIA who attended drug and alcohol classes at the request of friends, family, or an agency exhibited higher odds of completing SUD treatment programs. Encouragement and motivation from family and friends can induce higher self-esteem, increased self-efficacy, and less distress [19].

Individuals who do not actively seek treatment are not as likely to reach a three-year remission and subsequently relapse [2, 8]. Receiving no intervention may incite addictive behavior and increase criminal activity [33]. To reduce recidivism, an increase in accessibility and high levels of motivation are necessary for individuals seeking SUD treatment [1, 24]. Higher rates of completion are seen among JIA with high levels of optimism and strong support networks [34]. Completing SUD treatment has been associated with higher rates of remission, which can provide physical and mental well-being in the long-term [1, 9].

### Limitations

The study had limitations that provide context for discussing interpretations and recommendations. The cross-sectional data limited the ability to establish either causal conclusions or the exact temporal sequence between the substance abuse, assigned treatment completion, and SU remission. Data reported by JIA to FLDJJ staff presents the potential for social desirability among JIA and limits ability to establish causal conclusions.

The sample represents Florida JIA, and the findings may not be generalizable to JIA in other populations. Likewise, the large number of variables considered in this study increases the chances of a Type I error. SU included non-prescription drugs, which prevents an analysis of the potential differences between drug types. Specifically, opioid use was a limited dependent variable, with less than 3 % reporting current opioid use. The Firth approach indicated that the limited variation was not problematic, however, community samples with larger samples of current users may uncover important nuances among users.

### Future directions

Future directions should explore the racial disparities in SUD treatment completion within the juvenile justice system. This investigation demonstrates disproportionate rates of incarceration among people of color and SUD treatment completion rates among people of color may be undermined due to this investigation’s expanded focus. Moreover, studies should be conducted with an emphasis on racial disparities to identify and evaluate the differences in the health outcomes among JIA.

Studies should also stratify the influences self-stigma and general public stigma have on SUD treatment completion. Studies that examine perceived stigma, closeness, contact with family members, and variation of treatment programs may advance the literature that can improve SUD treatment completion. Additionally, studies that analyze the potential differences of depression, anxiety, and various drug types on SUD treatment outcomes can improve the current approaches used to address specific needs of JIA.

In this investigation, longitudinal data was not assessed and the changes in the impact of SUD treatment completion in regard to the correlates of interest over time could not be explored. Furthermore, longitudinal investigations with nationally representative samples are necessary to develop effective treatment programs for JIA.

## Conclusion

In this investigation, we identified the factors associated with a high likelihood of SUD treatment completion among JIA in order to decrease the risk of relapse, recidivism, and adolescent mortalities. The results of the study show that JIA with increased treatment exposure, access to SUD treatment programs, and strong support networks have higher odds of adherence. Using the data collected in this investigation, the FLDJJ is expected to identify unmet substance use service needs among juvenile offenders and develop organizational and policy changes to combat highly widespread adolescent substance use and increasing adolescent fatalities.

## Data Availability

All datasets generated and analyzed are not publicly available but are available from the corresponding author upon reasonable request.

## Abbreviations

ACE: Adverse Childhood Experiences
AOR: At Odds Ratio
CI: Confidence Interval
FLDJJ: Florida Department of Juvenile Justice
JIA: Justice-Involved Adolescents
PACT: Positive Assessment Change Tool
SU: Substance Use
SUD: Substance Use Disorder
